# Myoma praevia and pregnancy

**DOI:** 10.11604/pamj.2019.33.216.14898

**Published:** 2019-07-17

**Authors:** Yousra Krimou, Sanae Erraghay, Ahmed Guennoun, Nisrine Mamouni, Chahrazad Bouchikhi, Abdelaziz Banani

**Affiliations:** 1 Department of Obstetrics and Gynecology I, University Hospital Center Hassan II, Fez, Morocco

**Keywords:** Myoma, pregnancy, praevia, miscarriage, dystocia, hemorrhage

## Abstract

The association of myoma and pregnancy is becoming more frequent due to the increasing age of first pregnancy. It may affect the outcome of fertility, pregnancy, labor and peripartum course. A 37 years old patient was referred to our unit for discovering uterine leiomyoma at 37 weeks of pregnancy. Ultrasound screening showed a praevia isthmic leiomyoma measuring 16cm. A caesarean delivery was scheduled and a large interstitial isthmic uterine myoma measuring 25cm was found. Hysterectomy was corporeal. The post-operative and puerperium course was normal.

## Introduction

Uterine fibroma disease is very common with an overall incidence of 40% to 60% by age 35 and 70% to 80% by age 50. The precise etiology of uterine fibroids remains unclear. It is associated with pregnancy in 0.5 to 4% of cases. It may affect the outcome of fertility, pregnancy, labor and peripartum course.

## Patient and observation

**Figure 1:**
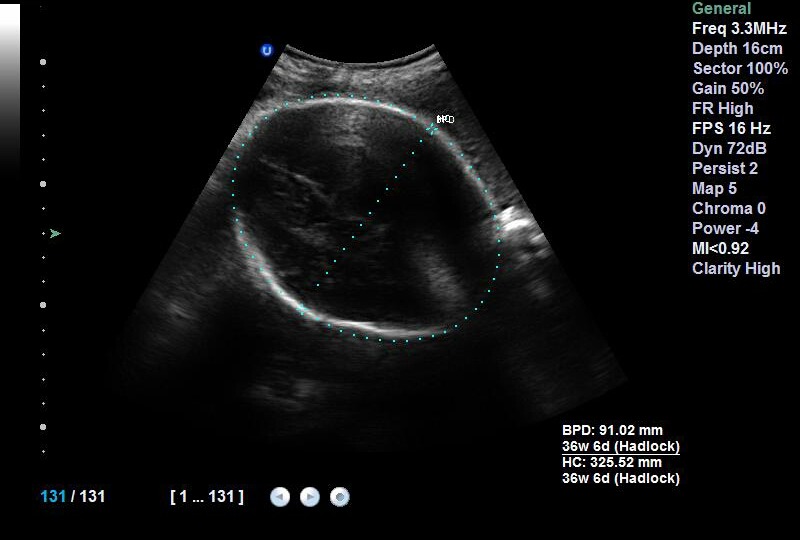
Sonographic image of myoma

**Figure 2:**
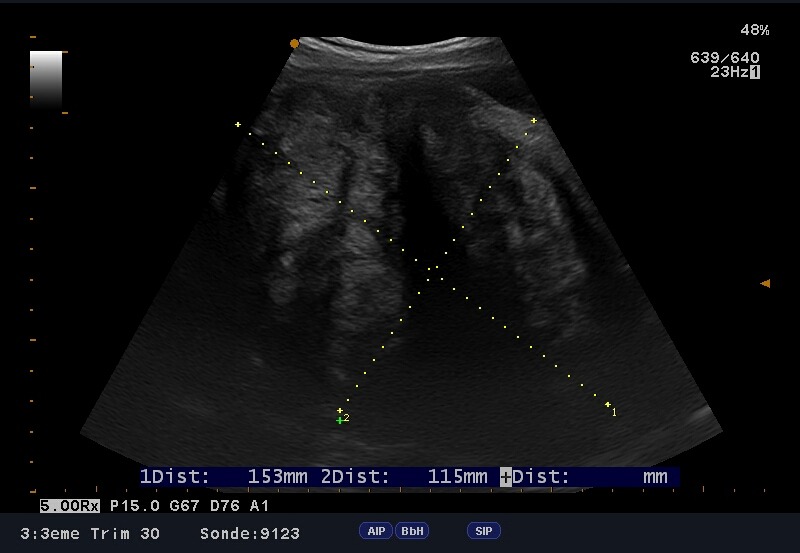
Sonographic image of fetal measurement

**Figure 3:**
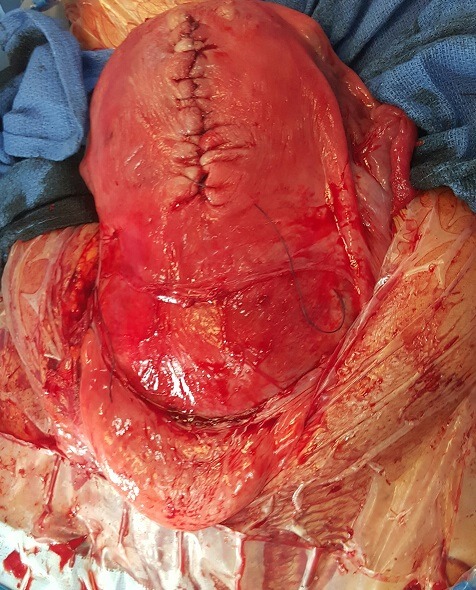
Per operative image of isthmic myoma

The patient A.B. is 37 years old, known for having a moderate asthma treated with on demand bronchodilator therapy. It is a first spontaneous pregnancy. Pregnancy wasn't followed and had an apparently normal course. Patient was referred to our unit for having uterine leiomyoma at 37 weeks of pregnancy. The obstetrical examination found an exaggerated uterine height at 38cm and an empty pelvic excavation. Ultrasound screening showed a praevia isthmic leiomyoma measuring 16cm (Figure 1); fetal biometry measurements were eutrophic (Figure 2) and placental insertion was fundic. Patient was immediately hospitalized in our unit; received corticosteroids for lung fetal maturation and a caesarean delivery was scheduled 24 hours later. Skin incision was median with the per operative discovery of a large interstitial isthmic uterine myoma measuring 40cm (Figure 3). Hysterectomy was corporeal. The post-operative and puerperium course was normal with a home return at the fifth day.

## Discussion

Leiomyomas are benign smooth muscle cell tumors of the uterus. Their prevalence varies according to ethnicity, age of the patients and techniques used to detect them. The association of myoma and pregnancy is becoming more frequent due to the increasing age of first pregnancy (currently close to 30 years). Myomas are formed in the majority of cases of smooth, well-organized muscle cell bundles. These fusiform muscle cells are usually homogeneous. Their mitosis rate is usually low. Degenerative changes can occur in fibroids such as hyaline fibrosis (60% of cases) or edema (50% of cases). Some fibroid subtypes have been described as mitotically active fibroids, cellular fibroids, hemorrhagic cell fibroids, atypical or “bizarre” fibroids and epithelioid fibroids. These different types of fibroids contain either a high level of cells or cells with a large nucleus and should be distinguished from leiomyosarcomas. The transformation mechanisms of the myometrium into fibroma include genetic anomalies [1], local growth factors and especially the action of estrogens and progesterone [2]. Diagnosis of fibroids in pregnancy is neither simple nor straightforward. Only 42% of large fibroids (5cm) and 12.5% of smaller fibroids (3-5cm) can be diagnosed on physical examination. Most fibroids are asymptomatic, 61% of myomas are discovered fortuitously on the first trimester ultrasound, others discovered in the occurrence of pelvic pain (13%); bleeding (11%) and exaggerated uterine height (3%). Prospective studies using ultrasound to follow the size of uterine fibroids throughout pregnancy have shown that 60%-78% of fibroids do not demonstrate any significant change in volume during pregnancy [3, 4]. Of the 22% to 32% of fibroids that did increase in volume, the growth was limited almost exclusively to the first trimester, especially the first 10 weeks of gestation, with very little if any growth in the second and third trimesters. The mean increase in volume in these studies was only 12,6% and the maximum growth was only 25% of the initial volume. Some studies have shown that small fibroids are just as likely to grow as large fibroids, whereas other studies have suggested that small and large fibroids (6 cm) have different growth patterns in the second trimester (small fibroids grow whereas large fibroids remain unchanged or decrease in size), but all decrease in size in the third trimester [4]. The majority of fibroids show no change during the puerperium, although 7.8% will decrease in volume by up to 10%. Approximately 10% to 30% of women with uterine fibroids develop complications during early and late pregnancy [5]. The main ones are reported in Table 1. Spontaneous miscarriage rates are greatly increased in pregnant women with fibroids compared with control subjects without fibroids (14% vs 7.6%, respectively) [5]. It is caused by increased uterine irritability and contractility, the compressive effect of fibroids, and compromise to the blood supply of the developing placenta and fetus. Size of the fibroid does not affect the rate of miscarriage, but multiple fibroids may increase the miscarriage rate; compared with the presence of a single fibroid only (23.6% vs 8.0%) [5]. The location of the fibroid may also be important. Early miscarriage is more common in women with fibroids located in the uterine corpus than in the lower uterine segment [6] and in women with intramural or submucosal fibroids. Bleeding in early pregnancy is significantly more common if the placenta implants close to the fibroid compared with pregnancies in which there is no contact between the placenta and fibroid (60% vs 9%, respectively) [7]. Pregnant women with fibroids are significantly more likely to develop preterm labor and to deliver preterm than women without fibroids (16.1% vs 8.7% and 16% vs 10.8%, respectively; Table 1) [8]. Multiple fibroids and fibroids contacting the placenta appear to be independent risk factors for preterm labor. In contrast, a recent systematic review suggests that fibroids are associated with a decreased risk of PPROM [8] (Table 1). The risk of placental abruption increases in women with fibroids (Table 1) [8]. Submucosal fibroids, retroplacental fibroids and fibroid' volumes 200 cm^3^ are independent risk factors for placental abruption. One retrospective study reported placental abruption in 57% of women with retroplacental fibroids in contrast with 2.5% of women with fibroids located in alternate sites [9]. One possible mechanism of placental abruption may be diminished blood flow to the fibroid and the adjacent tissues which results in partial ischemia and decidual necrosis in the placental tissues overlaying the leiomyoma [9].

**Table 1:** Cumulative risk of adverse obstetric outcomes in pregnant women with fibroids

	Exposed woman %	Non exposed woman %	OR	IC	p
Hospitalization	28.2	11.5	3.03	[1.65-5.56]	<0.001
Spontaneous miscarriage	3	0.9	3.2	[0.6-0.9]	<0.001
First trimester Bleeding	7.6	4.7	0.6	[0.5-0.7]	<0.001
Abruption	3	0.9	3.2	[2.6-4]	<0.001
PPROM	9.9	13	0.8	[0.6-0.9]	0.003
IUGR	11.2	8.6	1.4	[1.1-1.7]	<0.001
Anemia	26.6	7.4	4.37	[2.20-8.71]	<0.001
Preterm labor	16.1	8.7	1.9	[1.5-2.3]	<0.001
Preterm delivery	16	10.8	1.5	[1.3-1.7]	<0.001
Placenta prævia	1.4	0.6	2.3	[1.7-3.1]	<0.001

Fetal growth does not appear to be affected by the presence of uterine fibroids. Although cumulative data and a population-based study suggested that women with fibroids are at slightly increased risk of delivering a growth-restricted infant [10] Table 1. Rarely, large fibroids can compress and distort the intrauterine cavity leading to fetal deformities, such as: dolichocephaly, torticollis, and limb reduction defects. Numerous studies have shown that uterine fibroids are a risk factor for cesarean delivery [10, 11] and it is due to either the multiplicity of fibroids, large fibroids >5 cm, submucosal fibroids and fibroids in the lower uterine segment or fetal malpresentation (breech) or labor dystocia. However, the presence of uterine fibroids, even large fibroids (5 cm) should not be regarded as a contraindication to a trial of labor. The most frightening peripartum complications of myomas are retained placenta, post-partum hemorrhage and puerperal sepsis. Postpartum hemorrhage is significantly more likely in women with fibroids compared with control subjects (2.5% vs 1.4%, respectively). Fibroids may distort the uterine architecture and interfere with myometrial contractions leading to uterine atony and postpartum hemorrhage. This same mechanism may also explain why women with fibroids are at increased risk of puerperal hysterectomy. Only one study reported that retained placenta was more common in women with fibroids if it was located in the lower uterine segment [12]. Uterine rupture after abdominal myomectomy is extremely rare. In a retrospective study of 120 women delivering at term following abdominal myomectomy in which the uterine cavity was not entered, there were no cases of uterine rupture reported [13]. However, numerous case reports and case series describing intrapartum uterine rupture after laparoscopic myomectomy [14, 15]. Recent data suggest that such uterine ruptures occur prior to the onset of labor at the site of the prior laparoscopic myomectomy [16]. The absolute risk of uterine rupture following laparoscopic myomectomy remains low at 0.5% to 1%. It is rare for fibroids to be treated surgically in the first half of pregnancy. If necessary, however, several studies have reported that antepartum myomectomy can be safely performed in the first and second trimester of pregnancy [17-19]. Indications include intractable pain from a degenerating fibroid especially if it is subserosal or pedunculated, a large or rapidly growing fibroid, or any large fibroid (5 cm) located in the lower uterine segment. Obstetric and neonatal outcomes in women undergoing myomectomy in pregnancy are comparable with that in conservatively managed women, although women who had a myomectomy during pregnancy were far more likely to be delivered by cesarean due to concerns about uterine rupture. Although not supported by all studies, most authorities agree that every effort should be made to avoid performing a myomectomy at the time of cesarean delivery due to the well-substantiated risk of severe hemorrhage requiring blood transfusion, uterine artery ligation and/or puerperal hysterectomy. Myomectomy at the time of cesarean delivery should only be performed if unavoidable to facilitate safe delivery of the fetus or closure of the hysterotomy. Pedunculated subserosal fibroids can also be safely removed at the time of cesarean delivery without increasing the risk of hemorrhage. Uterine artery embolization (UAE) is used as an alternative procedure for treating large symptomatic fibroids in women who are not pregnant and most importantly, do not desire future fertility. A recent prospective study reported that UAE performed immediately after cesarean delivery in women with uterine fibroids may be effective in decreasing postpartum blood loss and minimizing the risk of myomectomy or hysterectomy by inducing shrinkage of the fibroids. Although not recommended, there are several reports of successful and uneventful pregnancies after UAE for uterine fibroids [19].

## Conclusion

Uterine fibroid is a very common finding in women of reproductive age. Majority of fibroids do not change their size during pregnancy, but one-third may grow in the first trimester. Although the data are conflicting and most women with fibroids have uneventful pregnancies, the weight of evidence in the literature suggests that uterine fibroids are associated with an increased rate of spontaneous miscarriage, preterm labor, placenta abruption, malpresentation, labor dystocia, cesarean delivery and postpartum hemorrhage.

## Competing interests

The authors declare no competing interests.
